# Fanconi Anemia Pathway Activation by FOXM1 is Critical to Bladder Cancer Recurrence and Anticancer Drug Resistance

**DOI:** 10.3390/cancers12061417

**Published:** 2020-05-30

**Authors:** Yun-Gil Roh, Jeong-Yeon Mun, Seon-Kyu Kim, Won Young Park, Mi-So Jeong, Tae Nam Kim, Won-Tae Kim, Yung Hyun Choi, In-Sun Chu, Sun-Hee Leem

**Affiliations:** 1 Department of Biological Science, Dong-A University, Busan 49315, Korea; royunkil@gmail.com (Y.-G.R.); jeongyeonmun90@gmail.com (J.-Y.M.); dkwl523@hanmail.net (M.-S.J.); kimwt33@nate.com (W.-T.K.); 2 Personalized Genomic Medicine Research Center, Korea Research Institute of Bioscience and Biotechnology (KRIBB), Daejeon 34141, Korea; seonkyu@kribb.re.kr; 3 Department of Bioinformatics, KRIBB School of Bioscience, Korea University of Science and Technology, Daejeon 34113, Korea; 4 Department of Pathology, Medical Research Institute, Pusan National University Hospital, Busan 49241, Korea; arkray-hyde@hanmail.net; 5 Department of Urology, Medical Research Institute, Pusan National University Hospital, Busan 49241, Korea; bigman1995@hanmail.net; 6 Department of Biochemistry, Dong-eui University College of Korean Medicine, Busan 47227, Korea; choiyh@deu.ac.kr; 7 Genome Editing Research Center, Korea Research Institute of Bioscience and Biotechnology (KRIBB), Daejeon 34141, Korea

**Keywords:** bladder cancer, FOXM1, FANCD2, Fanconi anemia pathway, DNA repair, cancer recurrence

## Abstract

Although the 5-year survival rate of patients diagnosed with nonmuscle invasive bladder cancer (NMIBC) has reached 85%, more than 50% of patients suffer from frequent recurrences. To identify molecular targets associated with recurrence of NMIBC, we analyzed gene expression data and found that FOXM1 and FANCD2 were involved in recurrence. Therefore, we investigated how these genes were involved in the mechanism of recurrence and confirmed their usefulness as biomarkers. Investigation have shown that FOXM1 directly regulated the transcription of FANCD2, which is the key gene of the Fanconi anemia (FA) pathway. Depletion of FOXM1 resulted in DNA repair defects in the FA pathway and in decreased resistance to chemotherapy. Thus, the FANCD2-associated FA pathway activated by FOXM1 is an important mechanism involved in chemotherapy-related recurrence. In conclusion, FOXM1 and FANCD2 can be used as prognostic factors that are associated with high risk of recurrence and with anticancer drug resistance properties in NMIBC patients.

## 1. Introduction

Bladder cancer (BC) is the fifth most frequent cancer among men, with an estimated 549,393 new diagnoses and 199,922 BC deaths per year worldwide (2018) [1]. BC is classified into categories as nonmuscle invasive bladder cancer (NMIBC) or muscle invasive bladder cancer (MIBC) according to the presence or absence of muscle layer invasion [2]. More than 70% of patients are diagnosed with NMIBC and are grouped into low, intermediate, and high risk according to the EORTC (The European Organization for Research and Treatment of Cancer) risk stratification table [3,4]. Among the three groups, high-risk NMIBC (T1, with high grade/G3, and/or carcinoma in situ (CIS)) results in poor prognosis and additional intravesical bacillus Calmette-Guerin (BCG) and anticancer drug treatments (doxorubicin (DOX) and mitomycin C (MMC)) are recommended after surgery [5]. Despite these treatments, more than 50% of patients with NMIBC develop recurrence, and 10% to 30% of recurrent patients progress to MIBC [6]. These reports indicate that traditional indicators such as tumor grade, T stage, and CIS used in the diagnosis of BC patients are not sufficient [7]. It is essential that a new method be developed to complement the present diagnostic methods and provide insight into the molecular mechanism of recurrence. Yet, the precise mechanism of recurrence has not been clarified.

A recent hypothesis in recurrence is that small numbers of anticancer drug-resistant cancer cells in a tissue survive and proliferate to cause recurrence after chemotherapy [8,9]. To explore the biomarkers of recurrence in bladder cancer cell lines and bladder cancer tissues, we wanted to find the relationship between drug resistance and recurrence of bladder cancer. According to recent reports, anticancer drug resistance is caused by an increase in drug efflux, anti-apoptosis activity, and DNA damage repair [10,11]. Most of the current anticancer drugs used to treat cancer patients are designed to cause DNA damage and cell death [12]. However, because cancer cells use various DNA damage repair mechanisms to repair DNA damage and prevent cell death, the inhibition of DNA damage repair in cancer cells has emerged as an important issue for effective chemotherapy [12].

In previous studies, we found that patients with high expression of FOXM1 have a high risk of recurrence, suggesting FOXM1 could act as a recurrent biomarker [13]. FOXM1 is a transcription factor known to regulate the progression of the G2/M phase [14]. However, recent studies have shown abnormal overexpression of FOXM1 in various cancer tissues, such as those of the bladder, liver, prostate, brain, breast, lung, colon, pancreas, skin, cervix, ovary, and mouth [15]. FOXM1 affects the abnormal functions of cancer cells, including proliferation, cell cycle progression, apoptosis, angiogenesis, and DNA damage repair [16,17,18,19,20]. FOXM1 is associated with cancer metastasis, recurrence, and resistance to various chemotherapeutic drugs, such as DOX, epirubicin, and MMC [21,22]. FOXM1 is known to regulate the transcription of various DNA repair factors to increase homologous recombination (HR), nonhomologous end joining (NHEJ), base excision repair (BER), and mismatch repair [23]. These reports suggest FOXM1 as a DNA repair regulator associated with resistance to various chemotherapeutic agents and proliferation.

DNA interstrand crosslinking (ICL) is known to be repaired by the FA pathway, which is one of the DNA repair pathways [24]. MMC, which is used in chemotherapy for high-risk NMIBC patients, induces ICL and apoptosis, thereby eliminating cancer cells [25,26]. The FA pathway is a complex DNA recovery pathway known to recruit various DNA repair pathways, such as nucleotide excision repair (NER), homologous recombination repair (HRR), and translation synthesis (TLS) to restore ICLs [24]. FOXM1 has been reported to be involved in many DNA repair processes, but there have been few direct reports of the FA pathway. However, our previous study of gene expression profile analysis identified FOXM1 and FANCD2 as recurrent biomarkers of bladder cancer [13]. Among the FA pathway factors, FANCD2 is ubiquitinated by the FA core complex and is known as a key factor in completing ICL repair [24]. During the FA pathway, a total of 19 FA proteins work together, and the FANCD2–FANCI protein complex restores ICL in the cell cycle S phase [27]. Therefore, the expression of FANCD2 is expected to contribute to the resistance to MMC by ICL repair. However, the association of FANCD2 with FOXM1 and its transcriptional control mechanism is unclear.

Herein, we analyzed whether the transcription factor FOXM1 directly regulates the expression of FANCD2 and whether the increased expression of FOXM1 affects ICL repair by the FA pathway. We analyzed whether the transcription factor FOXM1 directly regulates the expression of FANCD2 and whether the expression of FOXM1 affects ICL recovery by the FA pathway. Through this study, we identified genes associated with NMIBC recurrence and chemotherapy resistance. These genes can be used as prognostic biomarkers for recurrence and anticancer drug resistance and have also revealed potential molecular mechanisms that could be the basis for developing new therapies.

## 2. Results

### 2.1. Correlation between FOXM1 and FANCD2 Expression and Prognosis in Patients with Superficial Bladder Cancer

**Figure 1:**
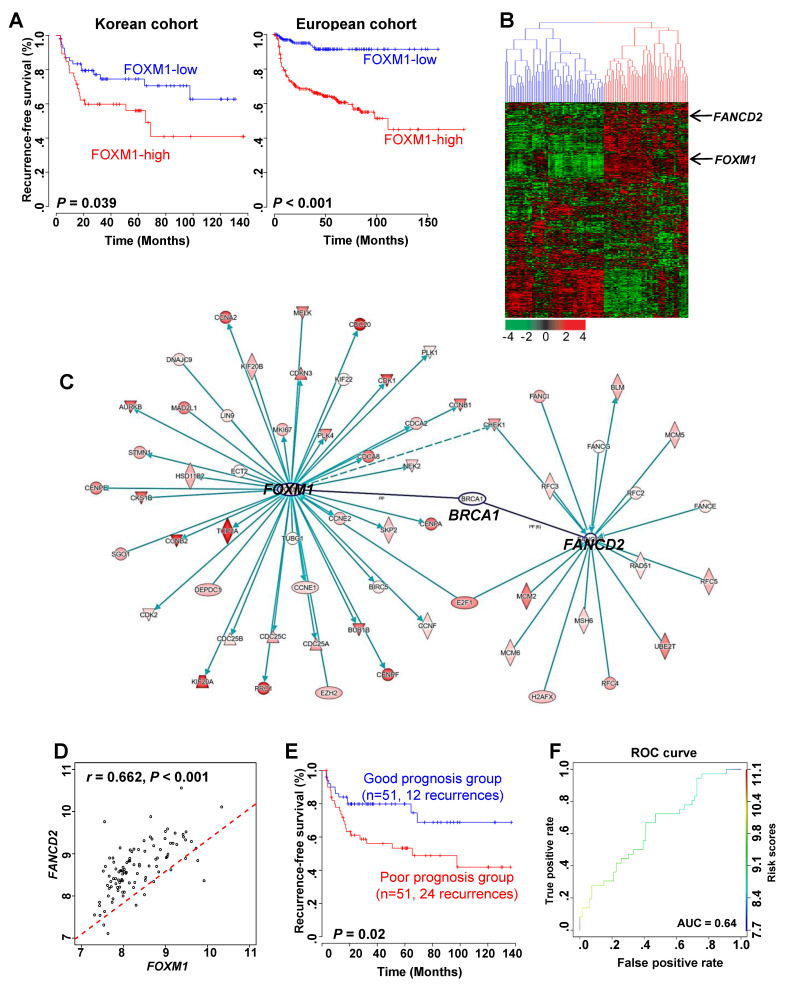
Recurrent prognosis and identification of genes associated with FOXM1 in NMIBC patients. (**A**) Recurrence-free survival (RFS) in the high and low FOXM1 expression groups in the Korean and Denmark cohorts. (**B**) Expression patterns of genes highly associated with FOXM1. A total of 509 genes that underwent cluster analysis had a high expression pattern with FOXM1 (r > 0.5) in the Korean cohort (*n* = 102). (**C**) Gene to gene network analysis of FOXM1-correlated genes in NMIBC using IPA (Ingenuity pathway analysis). (**D**) Correlation analysis of FOXM1 and FANCD2 in NMIBC in NMIBC patient gene expression data. (**E**) Prognosis of the combination of FOXM1 and FANCD2. Risk scores of two patient subgroups were calculated by expression levels of FOXM1 and FANCD2 with GSE13507 (Korean cohort). Prognosis indicates the RFS of NMIBC. (**F**) ROC (receiver operating characteristic) curve for prediction of recurrence using the FOXM1 and FANCD2 signatures.

In our previous studies, we showed that FOXM1 is highly associated with recurrence of NMIBC [13]. Therefore, we confirmed the link between recurrences in high and low FOXM1 expression groups. We found that recurrence was significantly increased in the high expression group of FOXM1 compared to the low expression group of FOXM1 in both Korean cohorts (GSE13507) and European cohorts (GSE5479) (Figure 1A). 

Next, we identified the genes associated with FOXM1 to determine the pathway through which the expression of FOXM1 affects recurrence. As a result, 509 genes were found to be associated with FOXM1 (Figure 1B). Hierarchical clustering analysis with 509 genes divided the NMIBC patients into two subgroups based on FOXM1 expression: a FOXM1-low cluster (Cluster 1) and a FOXM1-high cluster (Cluster 2) (Figure 1B). In addition, we divided the two groups according to the expression of FOXM1 into clinical factors to identify the recurrence prognosis. When the stage was divided into Ta and T1, recurrence significantly increased in the FOXM1-high group compared with the FOXM1-low group. Similarly, the same results were obtained when the grade was divided (Figure S1A,B).

We conducted a gene-to-gene network analysis based on the 509 genes associated with the expression of FOXM1. Then we performed a functional enrichment test and assessed the genes with Ingenuity Pathway Analysis. (Figure S2 and Table S1). As a result, FANCD2, which is known as a key factor in the FA pathway, was confirmed to be related to FOXM1 and is associated with its expression (Figure 1B,C). It was also confirmed that there was a significant positive correlation between FOXM1 and FANCD2 genes (Figure 1D). Risk scores of two patient subgroups were calculated using expression levels of FOXM1 and FANCD2 (Figure 1E). The area under the curve (AUC) by receiver operating characteristic (ROC) analysis was performed, and the proportion of recurrence-free survival in the good- and poor-prognosis groups was estimated in GSE13507 (Figure 1F). 

These results indicate that FOXM1 and FANCD2 may interact with each other and affect the recurrence of bladder cancer.

### 2.2. FOXM1 Directly Regulates the Transcription of the Key FA Pathway Gene, FANCD2

**Figure 2:**
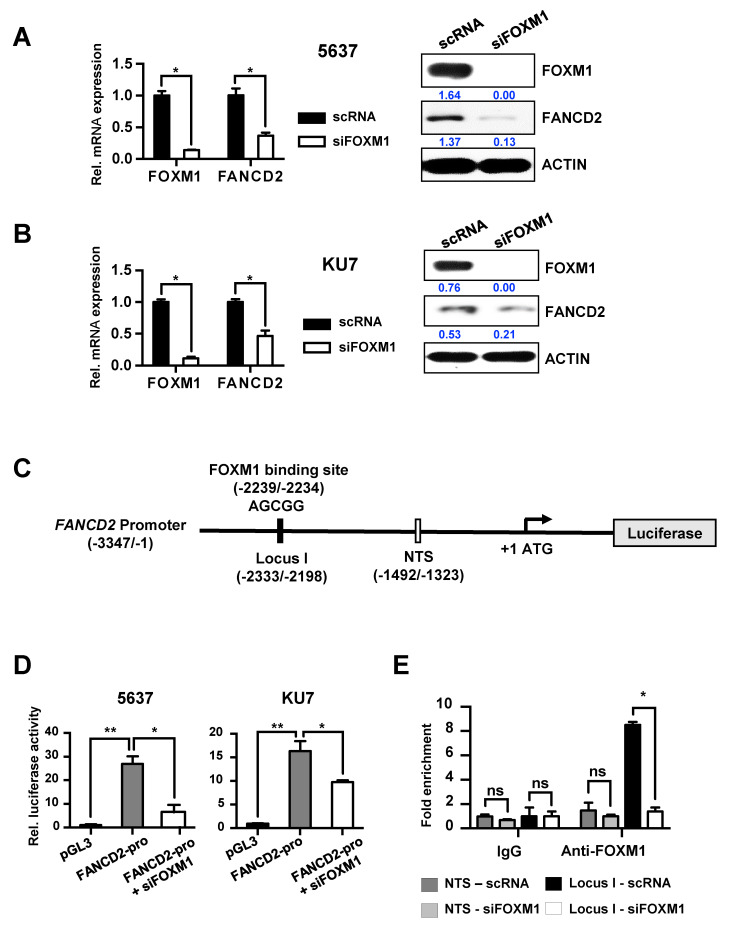
Direct transcriptional regulation of FANCD2 by FOXM1. (**A**) scRNA and siFOXM1 were transfected into 5637 and KU7 cells. mRNA expression was measured using qRT-PCR. (**B**) FOXM1 and FANCD2 protein expression was measured by Western blot in scRNA- and siFOXM1-transfected 5637 and KU7 cells. (**C**) A schematic diagram of the FANCD2 promoter region (−3347/−1). The black bar represents the putative FOXM1 binding site (−2239/−2234) and the qChIP amplification locus (−2333/-2198). The white bar represents the nontarget sequence of the qChIP amplification locus (−1492/−1323). (**D**) FANCD2 promoter activity was measured using a luciferase assay in siRNA (scRNA or siFOXM1)-transfected BC (Bladder cancer) cells. pGL3 vector was used as a control. (**E**) FOXM1 binding affinity of the FANCD2 promoter region. siRNA or siFOXM1 was transiently transfected into KU7 cells and immunoprecipitated using FOXM1 antibody and rabbit IgG (control). The amount of chromatin was measured using qRT-PCR along with a target site primer (I) and a nontarget site (NTS) primer. (*, *p* < 0.05; **, *p* < 0.01; and ns, not significant).

We investigated the modulation of FANCD2 expression by FOXM1 to determine the relationship between the two proteins. Inhibition of FOXM1 expression using siFOXM1 in both 5637 and KU7 bladder cancer cell lines significantly reduced FANCD2 expression at both the mRNA and protein levels (Figure 2A,B and Figure S3). Lentiviral particles containing a shRNA expression vector for FOXM1 or control NTS were transduced into KU7 and 5637 cells to produce stable FOXM1 knockdown (shFOXM1) and control (shNTS) cell lines. FANCD2 levels were reduced by knockdown of FOXM1, which was confirmed in both cell lines by qRT-PCR and Western blotting analysis (Figure S4A,B and Figure S5).

Next, we analyzed the sequence of the FANCD2 promoter region to determine whether the transcription factor FOXM1 binds to the promoter region of FANCD2 and directly regulates transcriptional activity. We identified one putative FOXM1 binding site in the promoter region of FANCD2 and constructed a luciferase vector with the FANCD2 promoter (Figure 2C). When this vector was transfected into the 5637 and KU7 bladder cancer cell lines, expression of the reporter vector was induced. However, additional siFOXM1 treatment significantly reduced the transcriptional activity of FANCD2 (Figure 2D). To further confirm that FANCD2 regulates the expression of FOXM1, FANCD2 was overexpressed or knocked out in the 5637 and KU7 cell lines. The results showed that FOXM1 expression was not altered, indicating that FANCD2 does not significantly affect FOXM1 (Figure S6A,B and Figure S7). These results show that the expression of FANCD2 is regulated by FOXM1. 

Immunoprecipitation was used to examine the FOXM1 binding affinity for the FOXM1 promoter region, which would reveal whether FOXM1 directly regulates FANCD2. Control siRNA or siFOXM1 were transfected into KU7 cells, and immunoprecipitation was performed using control IgG and FOXM1 antibodies. The result is shown in Figure 2E; we found that FOXM1 binds directly to the locus I region in the promoter of FANCD2. 

These results demonstrate that FOXM1 binds directly to the promoter region of FANCD2 and regulates its expression.

### 2.3. FOXM1 is Involved in Anticancer Drug Resistance Through the Direct Regulation of FANCD2

**Figure 3:**
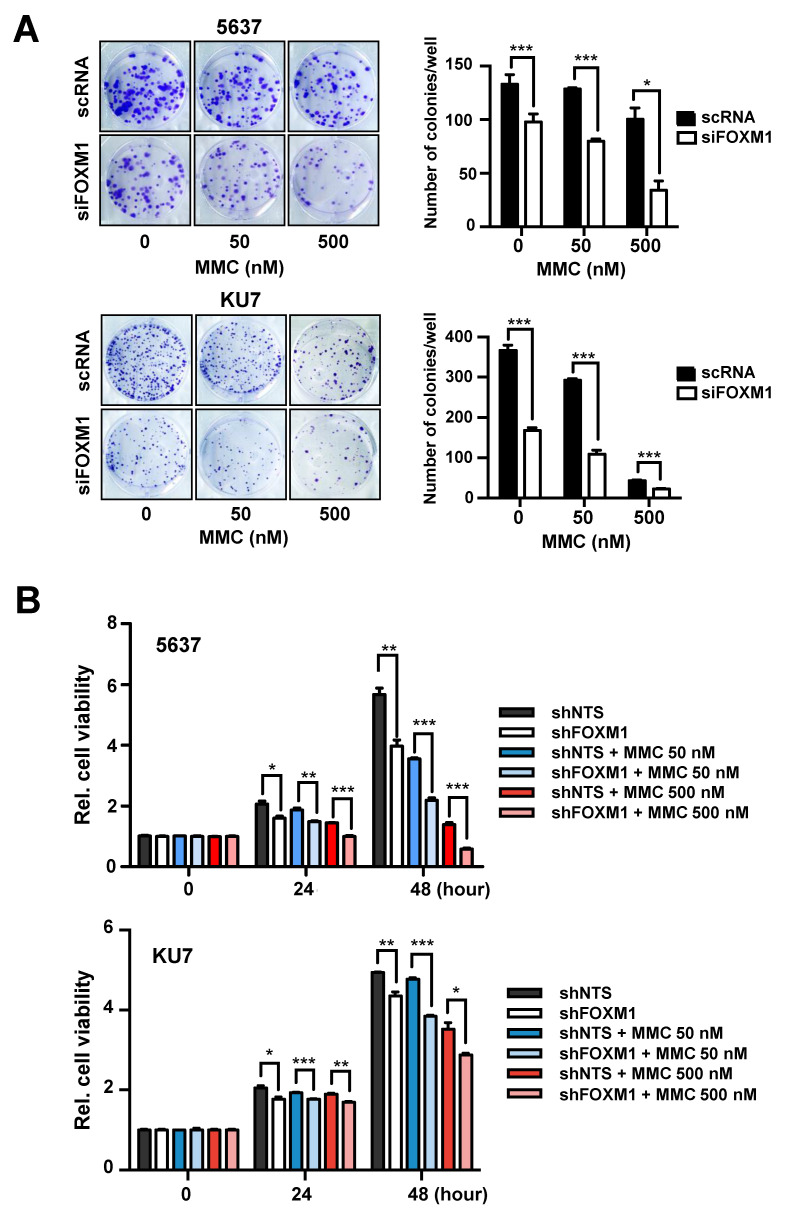
Analysis of MMC resistance following suppression of FOXM1 expression. (**A**) siRNA (scRNA or siFOXM1)-transfected 5637 and KU7 cells exposed to the indicated concentrations of MMC for 24 hours and seeded at 500 cells/well. After 10 days, colonies were stained with crystal violet and counted using a microscope. (**B**) The survival rate of 5637 and KU7 cells stably expressing shNTS or shFOXM1 was measured by MTT assay after exposure to the indicated concentrations of MMC (Mitomycin C) at the indicated times. (*, *p* < 0.05; **, *p* < 0.01; and *** *p* < 0.001).

Many anticancer drugs, including MMC, kill cancer cells by causing DNA damage. The ability of cancer cells to exhibit excessive activity of DNA repair mechanisms is considered to be a cause of cancer chemoresistance [10]. Therefore, we examined cell survival in cells transfected with control scRNA or siFOXM1 and in cells stably expressing shNTS or shFOXM1, and we also examined the correlation between MMC resistance and FOXM1 expression (Figure 3A,B). 5637 and KU7 cell lines were treated with various concentrations of MMC (0, 50, and 500 nM), and cell viability was examined by colony formation assay and MTT assay. The results revealed that when FOXM1 was inhibited, the survival rate of cells was remarkably decreased (Figure 3A,B).

FOXM1 suppression has been confirmed to reduce resistance to MMC, suggesting that FOXM1 is associated with DNA repair activities such as the FA pathway. From the above results, we confirmed that FOXM1 regulates the expression of FANCD2 (Figure 2) and that the expression of these two genes influences recurrence (Figure 1). We therefore investigated whether FOXM1 is involved in anticancer drug resistance through the direct control FANCD2, which is involved in the FA pathway. 

**Figure 4:**
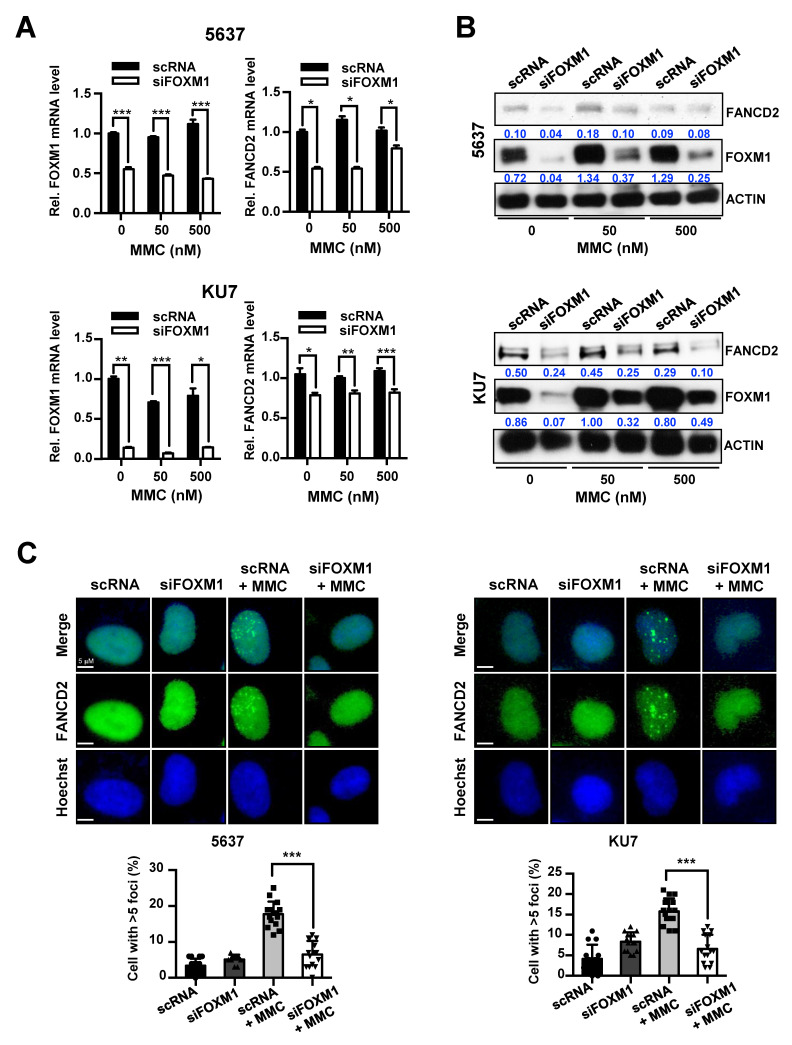
Control of FANCD2 expression and repair activity of FANCD2 following inhibition of FOXM1 and MMC treatment. siRNA (scRNA or siFOXM1)-transfected 5637 and KU7 cells were exposed to MMC for 24 hours. (**A**) FOXM1 and FANCD2 mRNA expression was measured by qRT-PCR. (**B**) FOXM1 and FANCD2 protein levels were analyzed by Western blot. (**C**) Nuclear foci formation of FANCD2 measured by immunofluorescence. Hoechst 33342 stained nuclear region. (*, *p* < 0.05; **, *p* < 0.01; and ***, *p* < 0.001).

We conducted experiments to determine whether there is a relationship between the expression of these two genes and resistance to anticancer drugs. First, we investigated whether the expression of FANCD2 is regulated by siFOXM1 treatment. Both cell lines were transfected with control scRNA or siFOXM1 and then were treated with various concentrations of MMC to confirm the reduction of FANCD2 expression by siFOXM1 (Figure 4). The expression of FANCD2 mRNA and protein decreased significantly following inhibition of FOXM1 in 5637 and KU7 cells that were treated with MMC (Figure 4A,B and Figure S8). Furthermore, an increase in cell viability due to FANCD2 overexpression was identified at 48 hours after the treatment. Cell viability increased when FANCD2 was overexpressed in the shFOXM1 cell line even after treatment with 50 and 500 nM MMC for 48 hours. (Figure S9A–C). 

FANCD2 is a key gene in the FA pathway, and the deletion of this gene affects the activity of DNA repair by the FA pathway [27,28]. Therefore, we determined how FOXM1, which directly affects DNA repair activity, regulated the expression of FANCD2. After we transfected bladder cancer cell lines with control scRNA or siFOXM1, MMC was used to treat cells, and FANCD2-foci were observed during the DNA repair process. The results showed that the formation of FANCD2-foci was significantly reduced by suppression of FOXM1 expression in 5637 and KU7 bladder cancer cell lines (Figure 4C). 

These results suggest that depletion of FOXM1 expression leads to a decrease in the expression of FANCD2, and consequently decreases the DNA repair response of FANCD2 to MMC. 

### 2.4. Expression of FOXM1 Affects DNA Repair by the FA Pathway 

The Fanconi anemia pathway is a mechanism to complete DNA repair by recruiting various DNA repair mechanisms such as nucleotide cleavage repair, mutagenesis, and homologous recombination. Therefore, to investigate whether FOXM1 directly affects the FA pathway, the activity of these various DNA repair mechanisms was measured after suppression of FOXM1 expression.

**Figure 5:**
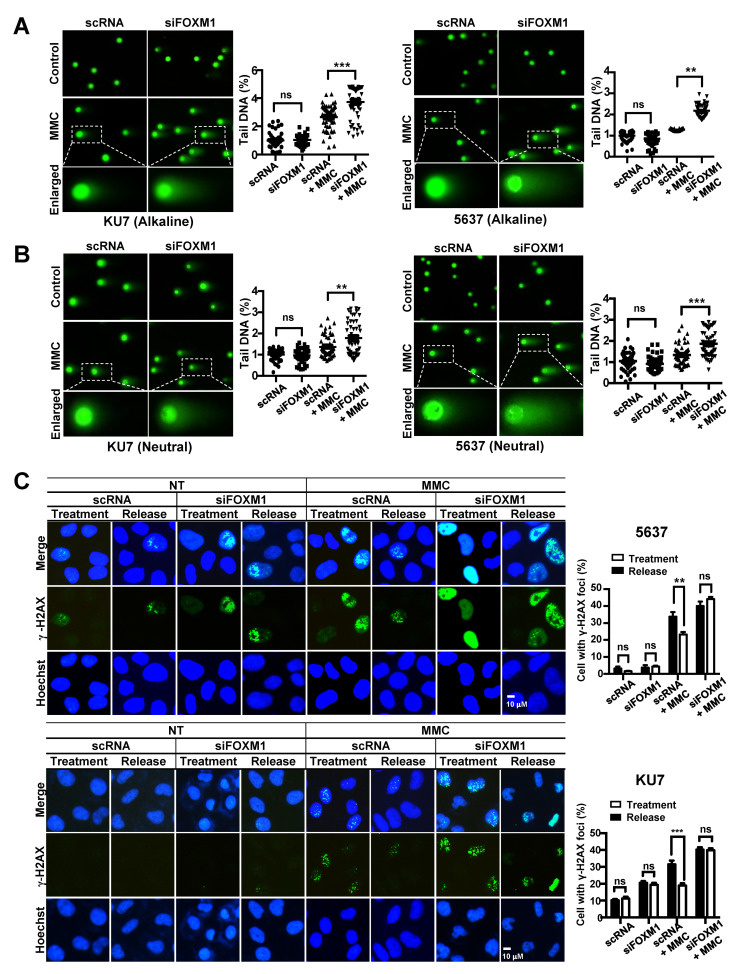

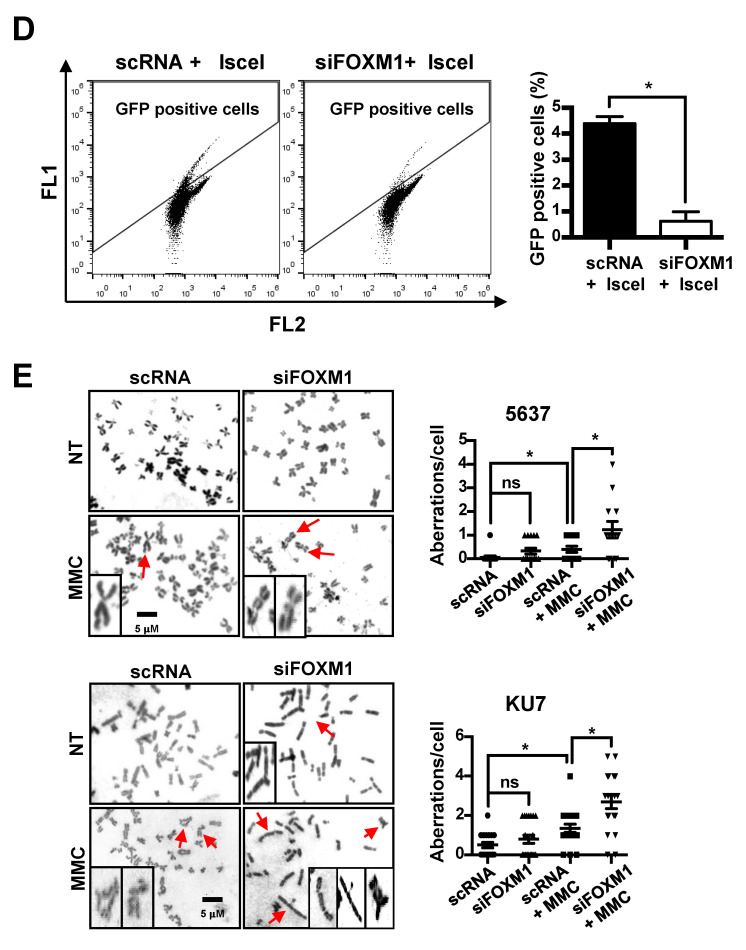
Measurement of DNA damage and repair activity in FOXM1-inhibited bladder cancer cells. To determine DNA damage and repair activity, siRNA (scRNA or siFOXM1)-transfected 5637 and KU7 cells were exposed to 0.5 μM MMC for 24 hours. A comet assay was performed to measure DNA damage. (**A**) The alkaline condition was used to measure single strand breaks. (**B**) The neutral condition was used to measure double strand breaks. γ-H2AX staining was performed to measure DNA recovery activity. (**C**) siRNA (scRNA or siFOXM1)-transfected 5637 and KU7 cells were exposed to 0.5 µM MMC for 4 hours. Then, the cells were incubated in fresh media for 24 hours. An HR assay was performed to measure HRR activity. (**D**) GFP (Green Fluorescent Protein)-positive cells were counted to measure HRR activity using FACS (Fluorescence-activated cell sorting) in siRNA (scRNA or siFOXM1) and I-sceI co-transfected U2OS-DRGFP cells. Chromosomal aberrations were analyzed to detect chromosomal abnormalities. (**E**) siRNA (scRNA or siFOXM1)-transfected 5637 and KU7 cells were exposed to 0.5 µM MMC for 24 hours, and chromosomal aberrations were measured by Giemsa staining. (*, *p* < 0.05; **, *p* < 0.01; *** *p* < 0.001; and ns, not significant).

We conducted a comet assay to investigate whether the inhibition of FOXM1 expression affects the DNA repair process of single strand breaks (SSBs) and double strand breaks (DSBs). DNA damage was induced by treating 5637 and KU7 BC cell lines with MMC, and the degree of DNA repair was measured by the tail length in a comet assay. Inhibition of FOXM1 expression increased comet tail length in both alkaline gels (DSBs and SSB-detectable) and neutral gels (DSB-detectable) (Figure 5A,B). These results show that the repair of SSBs and DSBs caused by treatment with MMC is decreased by suppression of FOXM1 expression (Figure 5A,B).

We also performed γ-H2AX staining to confirm that the suppression of FOXM1 reduces DNA damage repair. Cells were exposed to MMC for 4 hours, and γ-H2AX foci were measured after 0 hours (treatment) and 24 hours (release) of repair time (Figure 5C). DNA damage caused by MMC treatment was identified as γ-H2AX foci, and these foci were reduced by DNA repair after a 24-hour repair period in the scRNA control-treated cells (Figure 5C). On the other hand, it was confirmed that the siFOXM1 group remained, the number of γ-H2AX foci was over the same period of repair in 5637 and KU7 bladder cancer cell lines (Figure 5C). 

Additionally, we measured the homologous recombination repair (HRR) activity to determine whether the HRR involved in the last step of the Fanconi anemia pathway was regulated by the inhibition of FOXM1 expression. We found that the activity of HRR significantly decreased by inhibiting the expression of FOXM1 (Figure 5D).

Next, we measured chromosomal aberrations known to occur when there is a repair problem with the FA pathway. The effect of FOXM1 inhibition on chromosomal aberrations induced by MMC treatment was examined. The scRNA and siFOXM1 groups showed a similarly low number of chromosomal aberrations (Figure 5E). However, when treated with MMC, there was a significant increase in chromosomal aberrations in both groups, especially in the siFOXM1 group, compared with that of the scRNA control group (Figure 5E). This suggests that depletion of FOXM1 results in an inability to repair chromosomal aberrations to the level of the control.

Taken together, these results suggest that FOXM1 is involved in various DNA damage repair pathways associated with the FA pathway that are induced by MMC treatment. 

### 2.5. Verification of FOXM1 and FANCD2 as Biomarkers for Predicting the of Recurrence of Bladder Cancer

**Figure 6:**
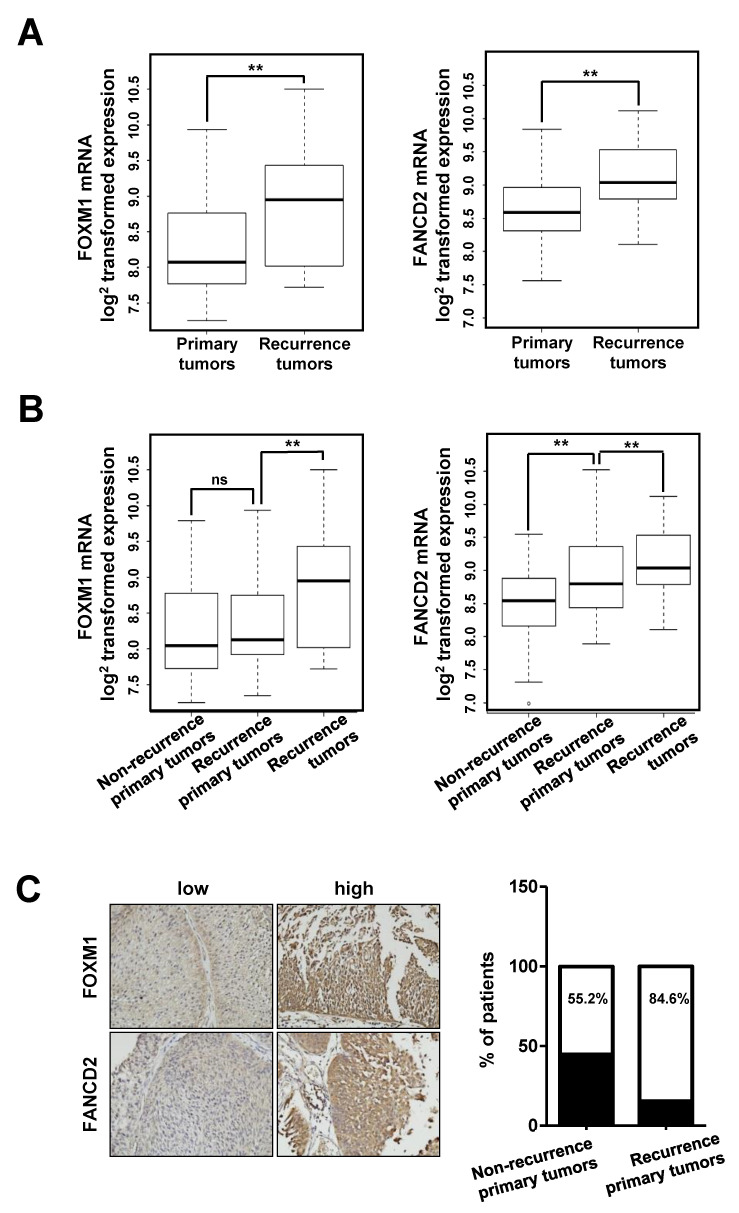
Expression of FOXM1 in NMIBC tissues. (**A**) Boxplot for two NMIBC patient groups (primary and recurrent tumor groups). (**B**) Boxplot for three NMIBC patient groups (nonrecurrent primary, recurrent primary, and recurrent tumor groups)**.** (**C**) RFS (Recurrence free survival) of FOXM1 expression levels in two independent NMIBC cohorts. (**, *p* < 0.01; and ns, not significant).

We then assessed whether FOXM1 and FANCD2 affect the recurrence of bladder cancer by analyzing their expression in clinical tissues. We examined the gene expression levels of FOXM1 and FANCD2 in primary and recurrent tumor tissues in the NMIBC patient group (Figure 6). Figure 6A shows the comparison between the primary and recurrent tumor groups in the NMIBC patient groups. The results showed that the expression of these genes was significantly higher in recurrent cancer tissues than it was in primary cancer tissues (*p* = 0.004; FOXM1 and *p* = 0.001; FANCD2 by two sample t-test, Figure 6A). 

To determine whether FOXM1 and FANCD2 were associated with recurrence according to the expression in primary cancer tissue, primary cancer tissue was classified into three NMIBC patient groups (no-recurrent primary, recurrent primary, and recurrent tumor groups), and the mRNA expression patterns were determined (Figure 6B). The results revealed that FOXM1 expression was not significantly different between the two primary tumor tissues, but FANCD2 expression was significantly higher in recurrent tumor tissues than it was in nonrecurrent tumor tissues (*p* = 0.486; FOXM1 and *p* = 0.002; FANCD2, Figure 6B). In the three-group classification, the expression of these two genes was significantly higher in recurrent cancer tissues than it was in primary cancer tissues (*p* = 0.003; FOXM1 and *p* = 0.02; FANCD2, Figure 6B).

Moreover, we determined the protein levels of FOXM1 and FANCD2 by IHC (Immunohistochemistry) in 57 bladder cancer samples (30 nonrecurrent primary tumors and 27 recurrent primary tumors) using a tissue microarray (Figure 6C). We identified 13 cases (44.8%) with low intensity FOXM1 or/and FANCD2 staining (IRS ≤ 3) and 16 cases (55.2%) with high intensity FOXM1 and FANCD2 staining (IRS > 3) in tissue microarray (TMA) samples of nonrecurrent tumors. In addition, there were 4 cases (15.4%) with low intensity FANCD2 or/and FANCD2 staining and 22 cases (84.6%) with high intensity FOXM1 and FANCD2 staining in the TMA of recurrent tumors (*p* = 0.018) (Figure 6C). Thus, the IHC results were correlated with clinicopathological and recurrence data. 

## 3. Discussion

Although NMIBC is known to have a relatively high survival rate compared to MIBC, the incidence rate is expected to significantly increase with the increase in the elderly population [25]. However, NMIBC has a high recurrence rate of over 50% and requires repeated treatment [7]. Therefore, the cost of the treatment is inevitably high, which may result in a negative effect on the quality of life of the elderly population [25]. Therefore, if biomarkers could be used to predict the recurrence of BC, accurate diagnosis and effective treatment could be conducted, which would prevent predicted recurrence problems.

Our previous studies have shown that recurrence significantly increases in NMIBC patients with high *CCNB1* expression [13]. In addition, BCG, which is currently used in high risk NMIBC treatment, requires a definite treatment indicator, according to the current treatment standards, because of the risk of adverse effects in some patients with no therapeutic responses or serious side effects [2]. Interestingly, our results showed that treatment with BCG can be more effective in patients with high *CCNB1* expression [13]. These results indicate that *CCNB1* is a suitable biomarker since *CCNB1* expression can also be used as a criterion to clearly indicate whether therapy should be performed [13]. However, the risk of side effects that may be caused by BCG still remain. The increased effect of BCG in the high *CCNB1* expression group was found as a result of demonstrating that immunotherapy is effective in a group of patients not responding to conventional chemotherapy [13]. Therefore, if this group can increase the effectiveness of chemotherapy, then safe and effective treatment of BCG is possible.

In this study, we identified FOXM1 as a driver gene that is strongly associated with bladder cancer recurrence and is known to regulate expression upstream of *CCNB1*. In addition, we could determine the recurrence rate of patients classified according to the expression of FOXM1 and FANCD2 (Figure 1 and Figure 2) and by using these genes as biomarkers in combination with *CCNB1* [13], we could predict the prognosis of patients with NMIBC after the first surgery. 

We also investigated the pathway through which FOXM1 acts to interfere with anticancer drug therapy and the mechanism of cancer recurrence. We examined the mechanism of the FANCD2 transcriptional regulation by FOXM1 (Figure 3) and demonstrated that MMC-induced DNA damage is reduced by the inhibition of FOXM1 (Figure 4 and Figure 5). The transcriptional regulatory mechanism of FANCD2, a key element of the FA pathway, was confirmed. It is expected that the effect of MMC on chemotherapy can be increased by confirming decreased DNA repair activity as a result of decreased FANCD2 expression. Currently, the expression of FANCD2 did not increase by MMC treatment because MMC repressed DNA replication by inhibiting DNA replication and transcription to eliminate cancer cells. Our results demonstrate that the inhibition of the expression of FOXM1, the driver gene, in NMIBC recurrence can inhibit different anticancer resistance pathways. Although new drugs targeting FOXM1 have not yet been developed, natural substances that restrict FOXM1 and inhibitors have been identified [26,29]. Therefore, it is expected that more effective chemotherapy will be achieved if these substances are used in concert with anticancer drugs. In addition, NMIBC treatment will be more effective if anticancer drugs targeting FOXM1 are developed.

Previous studies have shown that FOXM1 can regulate *CCNB1* expression to induce cell proliferation and recurrence [30]. The study also showed that FOXM1 can increase anticancer drug resistance by regulating the expression of FANCD2, which regulates the Fanconi anemia pathway and consequently increases DNA repair. Through this, we propose that FOXM1 directly regulates the expression of *CCNB1* and FANCD2 as an important pathway for recurrence and thereby is involved in cell proliferation and anticancer drug resistance.

In summary, we confirmed that FOXM1 can modulate the DNA repair pathway by directly regulating FANCD2 transcription and that it regulates resistance to MMC. Finally, in the case of FOXM1 and FANCD2, we confirmed that the expression could be used as a biomarker to predict recurrence and survival rates.

## 4. Materials and Methods

### 4.1. Cell Culture and Reagents

The 5637 and HEK-293T cells originated from the American Type Culture Collection (ATCC). KU7 and U2OS-DRGFP cells were provided by Ju-Seog Lee (The University of Texas MD Anderson Cancer Center, Houston, TX, USA). The 5637 cells were cultured in RPMI 1640 medium (HyClone, UT, USA) supplemented with 10% FBS (Fetal Bovine Serum, HyClone, UT, USA) and 1% penicillin/streptomycin (P/S, Gibco, NY, USA). HEK293T and KU7 cells were cultured in H-DMEM (HyClone) supplemented with 10% FBS and 1% P/S. The U2OS-DRGFP cells were grown in McCoy’s 5A (Modified) medium (Gibco, NY, USA), plus 10% FBS and 1% P/S. All cells were incubated at 37 °C under 5% CO_2_ in a humidified incubator. MMC, puromycin, and hexadimethrine bromide (polybrene) were purchased from Sigma (MO, USA) and were dissolved in sterile dH_2_O. 

### 4.2. Plasmid, Small-Interfering RNA, and Transfection

pGL3-Basic and pRL-Renilla luciferase reporter plasmids were purchased from Promega (WI, USA). To generate the pGL3 Basic-FANCD2 promoter (from −3347 to –1), human genomic DNA was amplified by PCR using the indicated primer sets (Table S2). We purchased shRNA for FOXM1 from Sigma. The plasmids that stably expressed a shRNA against *FOXM1* (shFOXM1) were established in a pLKO.1-TRC cloning vector (Addgene, MA, USA). We used a pLKO.1-puro nontarget shRNA plasmid (Sigma) for the negative control. pMD2.G and psPAX2 plasmids were purchased from Addgene (MA, USA). To construct pFANCD2 (FANCD2 overexpression vector), the coding sequence (CDS) of FANCD2 was inserted into the *Nhe*I/*Xho*I restriction enzyme sites of a pcDNA^TM^6/V5-His A plasmid (Invitrogen, MA, USA). All constructs were verified using a DNA sequencing. 

Scrambled RNA (scRNA) was purchased from Shanghai GenePharma (Shanghai, China). siFOXM1 (5’-GGACCACUUUCCCUACUUU-3’) was synthesized by the ST Pham Oligo Center (Korea). Plasmid and siRNA transfection were conducted using jetPRIME reagent (Polyplus, NY, USA) according to the manufacturer’s protocol.

### 4.3. Quantitative Real-Time PCR (qRT-PCR) and Luciferase Assay

All RNA was obtained from BC cells using RNAiso (TAKARA, Shiga, Japan) according to the standard protocol, and the synthesis of complementary DNA (cDNA) and qRT-PCR were performed using a PrimeScript RT reagent kit (TAKARA) according to the manufacturer’s instructions. cDNA was amplified using qRT-PCR with the indicated primer sets (Table S2) and CFX960 Optics Module (Bio-Rad, CA, USA). Amounts of mRNA were determined from the threshold cycle number with the expression of L19 as an endogenous control. All experiments were performed in triplicate and the values were averaged.

Luciferase assays were performed as described previously [31]. They were carried out using a Dual-Luciferase Reporter Assay System (Promega). To measure luciferase activity, a Wallac Victor 1420 Multilabel counter (PerkinElmer, MA, USA) was used. All firefly luciferase data were normalized to Renilla luciferase activities, each experiment was replicated three times, and the values were averaged.

### 4.4. Microarray

We analyzed a gene expression dataset from a previous study (GSE13507) that involved 165 primary Korean BC tissues. Among the 165 tissues, 102 tissues were identified as primary NMIBC. Clinical data including recurrence-free survival were acquired from the Chungbuk National University Hospital (Cheongju, South Korea). To estimate recurrence values of a signature combined with FOXM1 and FANCD2 genes, we selected a strategy that, for the genes in its signature, used the Cox regression coefficient (prognostic index (PI)). Ingenuity Pathway Analysis (IPA) was used to analyze the gene network-based activation regulator. Other gene expression datasets of BC patients with NMIBC from hospitals in the Swedish southern healthcare region (GSE32894; the SSH cohort, *n* = 213), Skane University Hospital (GSE32549; the SUH cohort, *n* = 92) and RNA sequencing data set (TCGA-BLCA; *n* = 412) were used to identify FOXM1 expressed genes. All gene expression datasets were opened at the National Center for Biotechnology Information (NCBI) Gene Expression Omnibus (GEO) database.

### 4.5. Generation of Stable Cell Lines

HEK-293T cells were transfected with pMD2.G (envelope plasmid), psPAX2 (packaging plasmid), and shRNA expressing plasmid (shNTS or shFOXM1) using jetPRIME reagent. The medium was changed 12 hours later and was harvested from the cells after 24 hours. The 5637 and KU7 cell lines were transduced with medium containing lentiviral particles and added polybrene was added. The cells stably expressing shRNA against NTS (shNTS) or FOXM1 (shFOXM1) were selected puromycin (5 μg/mL). FOXM1 mRNA and protein expression in cell lines was confirmed using qRT-PCR and Western blot assays.

### 4.6. Western Blot and Chromatin Immunoprecipitation (ChIP) Assay

Western blotting was performed as described previously [31]. Blots were conducted using the following antibodies: mouse-anti-β-Actin (A5441, Sigma), rabbit-anti-FOXM1 (Cat.A301-533A, Bethyl Laboratories, TX, USA), rabbit-anti-FANCD2 (Cat.NB100-182, Novus Biologicals, CO, USA). Horseradish peroxidase-linked goat anti-rabbit IgG polyclonal antibody (Cat.ADI-SAB-300-J, Enzo Life Sciences, NY, USA), and goat anti-mouse IgG polyclonal antibody (Cat.ADI-SAB-100-J, Enzo Life Sciences). Equal protein loading verified by detection of β-actin expression.

ChIP assays were performed as described previously [32]. Immunoprecipitation was performed using a rabbit-anti-FOXM1 antibody (Cat.A301-532A, Bethyl Laboratories, TX, USA). The indicated primer sets used for PCR amplification were a primer set for the FOXM1 site at position (Table S2).

### 4.7. Colony Formation Assay and MTT Assay

siRNA-transfected BC cells were seeded in 6-well plates at 1000 cells/well and then were incubated for 24 hours. Afterwards, the cells were treated with the indicated concentration of MMC for 24 hours. Then, the cells were harvested and reseeded in 6-well plates at 100 cells/well. After 2 weeks of incubation, colonies were fixed with 4% paraformaldehyde for 15 minutes at room temperature before being washed with PBS (Phosphate-buffered saline). Crystal violet (0.5%; Sigma) was used to stain the fixed cells for 30 minutes, which was followed by washing the plates with dH2O. The plates were then left to dry overnight. Colonies were counted using Carl Zeiss Axiovert 40 CFL microscopy (Göttingen, Germany).

MTT assays were performed as described previously [31]. Absorbance for each well was determined at 540 nm with a Wallac Vector 1420 Multilabel Counter (PerkinElmer, MA, USA). For each experimental condition, 3 wells were used.

### 4.8. Immunofluorescence

BC cells were seeded on coverslips coated with collagen (Sigma). Cells were treated both with 0.5 μM MMC, and control cells were untreated. After treatment, cells were washed with PBS and then fixed with 2% formaldehyde in PBS at room temperature (RT) for 20 minutes. After being washed with PBS, the process was followed by permeabilization with 0.5% Triton X-100 (Fluka, Buchs, Switzerland) in PBS at RT for 30 minutes. Then, cells blocked with 20% FBS were probed with the following antibodies in 5% FBS of PBS for 2 hours at RT: FOXM1 antibody (Cat.A301-533A, Bethyl Laboratories), FANCD2 antibody (Cat.NB100-182, Novus Biologicals, CO, USA), and phosphor-histone H2AX antibody (Cat.04-636, Millipore, MA, USA). After being washed with PBS, the cells were incubated with mouse and rabbit IgG-heavy and light chain antibodies (Cat.A90-116F, A120-101D4, Bethyl Laboratories) for 1 hour at RT and then were washed with PBS. Cells were costained with 100 μg/mL Hoechst 33342 and then were mounted with Vector Vectashield mounting media (Vector Laboratories, CA, USA). After fluorescence images were acquired using an LSM 700 confocal microscope (Carl Zeiss, Göttingen, Germany), the cells were counted in at least four randomly selected fields.

### 4.9. Comet Assay

BC cells (1 × 10^6^ cells) were harvested after treatment with 0.5 μM MMC for 24 hours and combined with molten LMAgarose (Trevigen, MD, USA). Before being incubated at 4 °C in lysis solution (Trevigen) overnight, cells were embedded in low melting agarose on a glass slide. Under alkaline conditions, slides were then immersed in an alkaline unwinding solution (200 mM NaOH, 1 mM EDTA, pH >13) in the dark at room temperature for 40 minutes before being placed in an electrophoresis slide tray. In addition, 850 mL of cold alkaline electrophoresis solution (200 mM NaOH, 1 mM EDTA, pH >13) was added, and a set power of 20 volts was applied for 30 minutes. Slides were first washed in dH_2_O twice for 5 minutes each and then in 70% EtOH for 5 minutes. Under neutral conditions, the slides were submerged in 1 × neutral electrophoresis buffer (10 × neutral electrophoresis buffer diluted 1:10 in dH_2_O 60.57 g Tris-Base, 204.12 g sodium acetate, pH = 9.0 with glacial acid) for 30 minutes. The slides were placed in an electrophoresis slide tray with 850 mL of cold 1 × neutral electrophoresis buffer, and a set power of 20 volts was applied for 45 minutes. The slides were then submerged in DNA precipitation solution (7.5 M NH_4_AC, 95% EtOH) for 30 minutes at room temperature. After they were washed with 70% EtOH for 30 minutes, they were dried at 37 °C for 15 minutes. Slides were then stained with SYBR Green for 30 minutes in the dark. Slides were rinsed in dH_2_O and finally were mounted using Vectashield mounting medium. Nuclei were visualized by fluorescence microscopy. The percentage of DNA was quantitated for 50 cells by using a microscope.

### 4.10. Homologous Recombination Assay and Chromosomal Aberration Assay

U2OS cells stably transfected with a DR-GFP plasmid vector were used for the HR assay. A total of 1 × 10^5^ siRNA-transfected cells were plated in a 6-well plate. Twenty-four hours later, the I-SceI endonuclease plasmid was delivered into the cells by transfection for 24 hours. Then, the cells were washed with PBS and harvested using trypsin. Then, cells expressing GFP were sorted by flow cytometry FC500 (Beckman Coulter, Krefeld, Germany) at 520 nm to analyze the efficiency of HR repair using the CXP v2.1 program.

For the detection of chromosomal aberration, siRNA transfected BC cells were treated with 0.5 μM MMC. Twenty-two hours later, the cells were exposed to 100 ng/mL colcemid (Sigma) for 2 hours. Then, the cells were treated with a hypotonic solution (75 mM KCl) for 20 minutes and then were fixed with 3:1 methanol/acetic acid. Slides were stained with a Giemsa solution (Sigma), and over 50 metaphase spreads were counted to detect aberrations. The relative number of chromosomal breaks and radials was calculated relative to scRNA or siFOXM1.

### 4.11. Immunohistochemistry (IHC) Staining and Antibodies

IHC was performed on a subset of 57 BC tissues from nonrecurrent primary cancer patients and recurrent primary cancer patients. A tissue microarray (TMA) was created from 30 nonrecurrent tumors, and 27 recurrent tumors were used. IHC was performed with a panel of antibodies against 2 markers (FOXM1 and FANCD2). All stained slides were digitalized with an SL801 autoloader and a Leica SCN400 scanning system (Leica Microsystems; Concord, Ontario, Canada) at a magnification equivalent to 20×. The images were subsequently stored in a Slide Path digital imaging hub (Leica Microsystems) at the Vancouver Prostate Centre. Values were assigned on a 4-point scale for each image. Descriptively, 0 represented no staining, 1 represented a low but detectable degree of staining, 2 represented a low detectable degree of staining, 3 represented clearly positive cases, and 4 represented strong expression. IHC was quantified for staining intensity (0–4).

### 4.12. Statistical Analysis

Data are represented as the mean ± SEM of three independent experiments. Unpaired Student’s t-tests were used to analyze the dissimilarities between the groups. Categorical data were analyzed by Fisher’s exact test. The cumulative recurrence was calculated by the Kaplan–Meier method and the log-rank test. Analyses were performed using GraphPad Prism 7 software (GraphPad Software, Inc., CA, USA). Asterisks, as described in the figure legends (ns, not significant; *, *p* < 0.05; **, *p* < 0.01; and ***, *p* < 0.001), were used to illuminate the statistically significant *p*-values that were less than 0.05. 

## 5. Conclusions

In this study, we confirmed the direct relevance of FOXM1 to the major FA pathway for anticancer drug resistance. These findings raised the possibility of using FOXM1-FANCD2 expression level as a prognostic factor when considering anticancer drug treatment and risk of recurrence in NMIBC patients.

## Supplementary Materials

The following are available online https://www.mdpi.com/2072-6694/12/6/1417/s1, Figure S1: Prognosis according to tumor stage and grade of two subgroups divided by FOXM1 expression in NMIBC patient cohort, Figure S2: Function enrichment test and network analysis, Figure S3: Uncropped blots showing all the western bands in 5637 and KU7 cell lines transfected with siFOXM1, Figure S4: The expression of FOXM1 and FANCD2 on knockdown stable cell lines, Figure S5: Uncropped blots showing all bands for stable knockdown of FOXM1 in KU7 and 5637 cell lines, Figure S6: No significance of the expression of FOXM1 by overexpressed FANCD2, Figure S7: Uncropped blots showing all the western bands in 5637 and KU7 cell lines transfected with pFANCD2 vector or siFANCD2, Figure S8: Uncropped blots showing all the western bands in 5637 and KU7 cell lines transfected with siFOXM1, treated to MMC, Figure S9: Increased cell viability due to overexpression of pFANCD2 in shFOXM1 cells, Table S1: Significantly enriched functions and their involved genes, Table S2: qPCR and plasmid construction primer set.
