# Supplementary material for: Fanconi Anemia Pathway Activation by FOXM1 is Critical to Bladder Cancer Recurrence and Anticancer Drug Resistance
Source: Cancers (Basel). 2020 May 30;12(6):1417. doi: 10.3390/cancers12061417 (PMC7352315; doi:10.3390/cancers12061417)
Supplement: Supplementary file 1 [file cancers-12-01417-s001.pdf]

cancers

MDPI

Supplementary Materials: Fanconi Anemia Pathway Activation by FOXM1 is Critical to Bladder Cancer Recurrence and Anticancer Drug Resistance

Table S1. qPCR and plasmid construction primer set.

Primer Name	Primer Sequence	Length	Product Size
qPCR L19 F	5′ GCG GAA GGG TAC AGC CAA T 3′	19 mer	76 bp
qPCR L19 R	5′ GCA GCC GGC GCA AA 3′	14 mer	
qPCR FOXM1 F	5′ TGC AGC TAG GGA TGT GAA TCT TC 3′	23 mer	157 bp
qPCR FOXM1 R	5′ GGA GCC CAG TCC ATC AGA ACT 3′	21 mer	
qPCR FANCD2 F	5′-ACA TAC CTC GAC TCA TTG TCA GT-3′	23 mer	248 bp
qPCR FANCD2 R	5′-TCG GAG GCT TGA AAG GAC ATC-3′	21 mer	
qChIP-FANCD2 I F	5′-AGC CCA GCT TAT TGC CTT CA-3′	20 mer	133 bp
qChIP-FANCD2 I R	5′-CGC CCG TAG TTT TCG ACT TT-3′	20 mer	
qChIP-FANCD2 NTS F	5′-GTA ACT ATG CTG GGC ATT AG-3′	20 mer	169 bp
qChIP-FANCD2 NTS R	5′-GTC ATC TTC CTT GAA AAG TG-3′	20 mer	
FANCD2 promoter F	5′-ATGC GAGCTC (*Sac*I) GGG TAT AGA GTA AGG CAA GGT-3′	32 mer	3367 bp
FANCD2 promoter R	5’-ATGC AAGCTT (*Hind*III) TTT GAC CAA TGT CTT GTG CAC-3′	30 mer	

Table S2. Significantly enriched functions and their involved genes.

Category	Functions	*p*-Value	#Molecules	Molecule List
Diseases and Disorders	Cancer	2.71E-25	212	*CBX7,TRAIP,HMMR,CCNB2,GPSM2,CENPE,CSE1L,MTHFD2,ADA,KNSTRN,TYMS,ASF1B,APOBEC3B,CCNE2,CDCA8,ESPL1,ACACB,TPM3,OTX1,SKP2,SKA3,IL17RB,CELSR3,TFRC,BYSL,ECT2,UBE2C,ANK3,KIF20A,NME1,PRDX1,TTI1,TTK,COL17A1,SNRPG,CDKN3,VRK1,SLC5A6,CXCR2,KIF2C,EXO1,NMU,ZNF695,TXNIP,TRIB3,BUB1,MAD2L1,CCNE1,CDKN2D,TMEM106C,FBXO5,KIF23,DLGAP5,PTTG1,SMC4,GALR2,CES2,SKA1,KLHDC1,ADAM28,SLC2A9,ERAL1,TUBA1C,ZWINT,B4GALT3,E2F2,KIFC1,C20orf27,KDM2B,ORC6,AURKA,BUB1B,ATAD2,CCNB1,YPEL5,KCNG1,PTPRN2,NDC80,FAM83D,TUBG2,SCD,CDC20,PCLAF,IL20RA,IRAK1,CDCA7,DNMT3B,CDC25B,RAD51,CCNA2,MICB,FANCD2,CFH,TRIP13,CDC25C,NUSAP1,RACGAP1,TUBG1,PRC1,FOXM1,BIRC5,TLR4,PCCA,MSH6,CKS1B,C16orf45,CENPF,MCM6,CDC7,MKI67,VWA5A,RBL1,CHAF1A,CTSV,PARPBP,ZBTB20,CEP250,DNMT1,FANCG,RCBTB2,UBE2S,RNASEH2A,ROR1,RBM28,CLCN5,RFC4,H2AFX,E2F1,LRP8,SGO1,NEK2,ARHGAP1,HIST1H1C,CDT1,POLQ,HMGB2,MTFR2,EIF4EBP1,CHEK1,NEIL3,ZBTB4,TOP2A,SLC19A1,MCM10,ORC1,C14orf159,CDC45,LETM1,CDC6,SPC25,DOT1L,CENPU,PLK1,NETO2,POLD1,CDK1,DHCR24,MCM2,CIRBP,KIF20B,CENPA,CD44,FEN1,HOXB2,INSIG1,XPO5,MELK,AURKB,PBK,STMN1,EZH2,FASN,BRCA1,HIST1H4H,CDC25A,MCM5,ANLN,HIST1H3C,GGH,KLHL3,TPX2,EBP,LAMA3,KIAA1524,PSMD4,TMPO,ABCC4,CDK2,KIF11,CEP55,RFC3,SRM,DDX39A,BCL2L12,WDHD1,ACOT7,HMOX1,TRIM24,PRIM1,ASPM,CIT,HSD11B2,CDCA5,TK1,UHRF1,CBX2,RFC5,LYAR,UBE2T,LPAR1,HOXC6,PAQR4*
	Organismal Injury and Abnormalities	2.71E-25	212	*CBX7,TRAIP,HMMR,CCNB2,GPSM2,CENPE,CSE1L,MTHFD2,ADA,KNSTRN,TYMS,ASF1B,APOBEC3B,CCNE2,CDCA8,ESPL1,ACACB,TPM3,THOP1,OTX1,SKP2,SKA3,IL17RB,CELSR3,TFRC,BYSL,ECT2,UBE2C,ANK3,KIF20A,NME1,PRDX1,TTI1,TTK,COL17A1,SNRPG,CDKN3,VRK1,SLC5A6,CXCR2,KIF2C,EXO1,NMU,ZNF695,TXNIP,TRIB3,BUB1,MAD2L1,CCNE1,CDKN2D,TMEM106C,FBXO5,KIF23,DLGAP5,PTTG1,SMC4,GALR2,CES2,KLHDC1,ADAM28,SLC2A9,ERAL1,TUBA1C,ZWINT,B4GALT3,E2F2,KIFC1,C20orf27,KDM2B,ORC6,AURKA,BUB1B,ATAD2,CCNB1,YPEL5,KCNG1,PTPRN2,NDC80,FAM83D,TUBG2,SCD,CDC20,PCLAF,IL20RA,IRAK1,CDCA7,DNMT3B,CDC25B,RAD51,CCNA2,MICB,FANCD2,CFH,TRIP13,CDC25C,NUSAP1,RACGAP1,TUBG1,PRC1,FOXM1,BIRC5,TLR4,PCCA,MSH6,CKS1B,C16orf45,CENPF,MCM6,CDC7,MKI67,VWA5A,RBL1,CHAF1A,CTSV,PARPBP,ZBTB20,CEP250,DNMT1,FANCG,RCBTB2,UBE2S,RNASEH2A,ROR1,RBM28,CLCN5,RFC4,H2AFX,E2F1,LRP8,SGO1,NEK2,ARHGAP1,HIST1H1C,CDT1,POLQ,HMGB2,MTFR2,EIF4EBP1,CHEK1,NEIL3,ZBTB4,TOP2A,SLC19A1,MCM10,ORC1,C14orf159,CDC45,LETM1,CDC6,SPC25,DOT1L,CENPU,PLK1,NETO2,POLD1,CDK1,DHCR24,MCM2,CIRBP,KIF20B,CENPA,CD44,FEN1,HOXB2,INSIG1,XPO5,MELK,AURKB,PBK,STMN1,EZH2,FASN,BRCA1,HIST1H4H,CDC25A,MCM5,ANLN,HIST1H3C,GGH,KLHL3,TPX2,EBP,LAMA3,KIAA1524,PSMD4,TMPO,ABCC4,CDK2,KIF11,CEP55,RFC3,SRM,DDX39A,BCL2L12,WDHD1,ACOT7,HMOX1,TRIM24,PRIM1,ASPM,CIT,HSD11B2,CDCA5,TK1,UHRF1,CBX2,RFC5,LYAR,UBE2T,LPAR1,HOXC6,PAQR4*
	Gastrointestinal Disease	8.95E-23	125	*CENPF,CCNB2,MKI67,GPSM2,CHAF1A,PARPBP,CSE1L,KNSTRN,DNMT1,TYMS,ASF1B,CCNE2,CDCA8,FANCG,THOP1,UBE2S,RNASEH2A,SKA3,RFC4,E2F1,TFRC,LRP8,SGO1,BYSL,NEK2,ECT2,UBE2C,KIF20A,ANK3,HIST1H1C,NME1,PRDX1,POLQ,HMGB2,TTK,VRK1,CDKN3,NEIL3,CXCR2,TOP2A,MCM10,ORC1,LETM1,SPC25,CDC6,CENPU,PLK1,TRIB3,POLD1,CDK1,MAD2L1,CCNE1,CIRBP,CENPA,CD44,FEN1,KIF23,HOXB2,DLGAP5,INSIG1,PTTG1,XPO5,MELK,CES2,AURKB,PBK,FASN,EZH2,TUBA1C,ZWINT,BRCA1,HIST1H4H,B4GALT3,E2F2,KIFC1,MCM5,C20orf27,ANLN,HIST1H3C,GGH,ORC6,AURKA,ATAD2,TPX2,CCNB1,EBP,LAMA3,KIAA1524,FAM83D,TMPO,CEP55,CDK2,TUBG2,SCD,DDX39A,PCLAF,ACOT7,WDHD1,CDCA7,IRAK1,CDC25B,DNMT3B,RAD51,HMOX1,CCNA2,MICB,ASPM,HSD11B2,CDCA5,CFH,TK1,TRIP13,NUSAP1,PRC1,TUBG1,FOXM1,BIRC5,UBE2T,LYAR,TLR4,LPAR1,CKS1B,MSH6,HOXC6,PAQR4*

	Reproductive System Disease	1.20E-18	137	*MCM6,CBX7,TRAIP,HMMR,CDC7,CCNB2,MKI67,VWA5A,RBL1,CTSV,CSE1L,CENPE,MTHFD2,ZBTB20,CEP250,TYMS,ASF1B,APOBEC3B,CCNE2,ACACB,ESPL1,FANCG,TPM3,RCBTB2,OTX1,UBE2S,RBM28,SKP2,CLCN5,IL17RB,RFC4,H2AFX,E2F1,LRP8,NEK2,ECT2,UBE2C,KIF20A,HIST1H1C,NME1,CDT1,POLQ,TTI1,TTK,MTFR2,CHEK1,EIF4EBP1,CDKN3,ZBTB4,TOP2A,MCM10,KIF2C,EXO1,NMU,ZNF695,C14orf159,LETM1,TXNIP,SPC25,CDC6,PLK1,POLD1,CDK1,BUB1,CCNE1,CDKN2D,MCM2,DHCR24,CIRBP,CENPA,TMEM106C,CD44,FBXO5,FEN1,PTTG1,SMC4,MELK,CES2,AURKB,ADAM28,EZH2,FASN,ERAL1,TUBA1C,ZWINT,BRCA1,B4GALT3,KIFC1,E2F2,CDC25A,KLHL3,AURKA,BUB1B,ATAD2,TPX2,CCNB1,YPEL5,KCNG1,LAMA3,PTPRN2,KIAA1524,PSMD4,ABCC4,KIF11,CDK2,RFC3,TUBG2,CDC20,PCLAF,CDCA7,IRAK1,DNMT3B,CDC25B,CCNA2,HMOX1,PRIM1,MICB,FANCD2,CIT,CFH,TK1,TRIP13,CDC25C,UHRF1,RACGAP1,TUBG1,PRC1,RFC5,FOXM1,BIRC5,TLR4,PCCA,LPAR1,CKS1B,MSH6,PAQR4,C16orf45*
	Endocrine System Disorders	4.26E-11	43	*HIST1H1C,PCLAF,HMMR,POLQ,SMC4,CCNB2,CES2,MTFR2,EIF4EBP1,CHEK1,CDKN3,FANCD2,CENPE,MTHFD2,TOP2A,TUBA1C,CFH,BRCA1,E2F2,ZNF695,TYMS,LETM1,TXNIP,RACGAP1,TUBG1,TPM3,FOXM1,BIRC5,TPX2,CDK1,CCNB1,TLR4,CDKN2D,DHCR24,H2AFX,E2F1,MSH6,CD44,LRP8,ECT2,CDK2,TUBG2,UBE2C*
Molecular and Cellular Functions	Cell Cycle	2.94E-36	129	*EME1,CENPF,TRAIP,CDC7,DSN1,CCNB2,GPSM2,RBL1,CHAF1A,PLK4,PARPBP,CSE1L,CENPE,DNMT1,LSM4,TYMS,ATG5,CCNE2,CDCA8,FANCG,MAD2L2,UBE2S,MASTL,SKP2,E2F1,TFRC,SGO1,NEK2,ECT2,UBE2C,NUDT1,KIF20A,NME1,CDT1,PLA2G10,TTI1,TTK,NUF2,SPAG5,CHEK1,EIF4EBP1,CDKN3,RFC2,TOP2A,SLC19A1,MCM10,KIF2C,EXO1,LETM1,RASSF6,TXNIP,SPC25,DOT1L,CDC6,PLK1,POLD1,CDK1,KIF22,BUB1,MAD2L1,CCNE1,CDKN2D,PDXP,KIF20B,CENPA,CD44,FBXO5,FEN1,RAD51AP1,KIF23,DLGAP5,PTTG1,SMC4,MELK,KNTC1,AURKB,LMNB1,STMN1,FASN,EZH2,ZWINT,BRCA1,KIFC1,E2F2,CDC25A,KIF14,STIL,ANLN,KDM2B,KIF4A,ORC6,RAD54L,AURKA,BUB1B,CDCA2,TPX2,CCNB1,KNL1,RECQL4,LIN9,NDC80,PKMYT1,CEP55,KIF11,CDK2,RFC3,CDC20,PCLAF,LSM2,DNMT3B,RAD51,CDC25B,CCNA2,HMOX1,FANCD2,UBIAD1,BLM,TRIP13,NUSAP1,TACC3,CDC25C,SNRPB,RACGAP1,FOXM1,BIRC5,CENPI,RASSF2,CKS1B,TONSL*
	Cellular Assembly and Organization	6.71E-18	90	*EME1,KIF23,DLGAP5,HMMR,PTTG1,SUV39H1,SMC4,CDC7,DSN1,CCNB2,MELK,AURKB,GPSM2,LMNB1,CHAF1A,STMN1,CSE1L,CENPE,ZWINT,BRCA1,KIFC1,DNMT1,KIF14,CDCA8,ESPL1,KIF4A,MAD2L2,ORC6,RAD54L,AURKA,ROR1,BUB1B,NDC1,TPX2,CCNB1,SKP2,EBP,TIMELESS,CCNF,NDC80,TMPO,SGO1,NEK2,ECT2,CDK2,KIF11,KIF20A,NME1,CDT1,POLQ,NUF2,TTK,WDHD1,PHLDB2,CHEK1,CDC25B,RAD51,CCNA2,DHCR7,FANCD2,CXCR2,CIT,CDCA5,BLM,KIF2C,CDC45,NUSAP1,CDC25C,TACC3,RACGAP1,PRC1,SPC25,DOT1L,CDC6,PLK1,FOXM1,POLD1,BIRC5,CDK1,BUB1,KIF22,TLR4,MAD2L1,CCNE1,PDXP,KIF20B,CENPA,MSH6,FEN1,TONSL*
	DNA Replication, Recombination, and Repair	6.71E-18	107	*TRAIP,SUV39H1,CDC7,DSN1,CCNB2,GPSM2,RBL1,CENPE,PARPBP,DNMT1,LSM4,TYMS,APOBEC3B,FANCG,MAD2L2,MASTL,CDCA4,SKP2,H2AFX,CCNF,E2F1,SGO1,NEK2,ECT2,KIF20A,CDT1,POLQ,TTI1,NUF2,EIF4G1,TTK,CHEK1,FANCE,TOP2A,RFC2,MCM10,KIF2C,EXO1,ORC1,LIG3,CDC45,RASSF6,CDC6,DOT1L,SPC25,PLK1,DEDD,POLD1,CDK1,KIF22,BUB1,MAD2L1,CCNE1,CDKN2D,FEN1,KIF23,DLGAP5,PTTG1,SMC4,MELK,AURKB,PBK,EZH2,ZWINT,BRCA1,KIFC1,CDC25A,KIF14,KIF4A,ORC6,RAD54L,AURKA,BUB1B,TPX2,CCNB1,TIMELESS,RECQL4,NDC80,TMPO,CDK2,KIF11,RFC3,CDC20,PCLAF,LSM2,RAD51,DNMT3B,CDC25B,HMOX1,CCNA2,FANCD2,PRIM1,BLM,NUSAP1,CDC25C,TACC3,SNRPB,RACGAP1,FOXM1,BIRC5,PPM1G,CENPI,CKS1B,MSH6,TONSL,UCK2,FANCI*
	Cellular Movement	8.15E-16	21	*KIF20A,KIF23,KIF14,NME1,NUSAP1,CDC20,ANLN,RACGAP1,KIF4A,AURKA,AURKB,BIRC5,MASTL,CCNB1,PDXP,KIF20B,TOP2A,NEK2,ECT2,CEP55,KIFC1*
	Cell Morphology	1.21E-12	49	*KIF23,PTTG1,RBL1,AURKB,LMNB1,CHAF1A,STMN1,FASN,BRCA1,KIFC1,DNMT1,KIF14,ATG5,KDM2B,KIF4A,MAD2L2,ORC6,TPM3,AURKA,TPX2,E2F1,LIN9,TMPO,NEK2,ECT2,KIF11,UBE2C,ANK3,KIF20A,PRDX1,NUF2,COL17A1,CHEK1,EIF4EBP1,KIF2C,TACC3,LETM1,RACGAP1,DOT1L,PLK1,FOXM1,BIRC5,INPP5D,KIF22,TLR4,MAD2L1,CCNE1,PDXP,CD44*
Physiological System	Tissue Morphology	1.81E-08	23	*TYMS,UHRF1,CDC20,RASSF6,KDM2B,RACGAP1,DOT1L,PRC1,CCNB1,SKP2,STMN1,CDC25B,HMOX1,CCNE1,KIF20B,E2F1,LAMA3,ST6GALNAC2,CD44,BRCA1,BLM,CDK2,CDC25A*

Development and Function	Organismal Survival	1.37E-06	19	*KIF23,TYMS,KIF14,CDCA8,MKI67,AURKB,FOXM1,BIRC5,HMOX1,CD44,TOP2A,LAMA3,SLC19A1,NDC80,CFH,BRCA1,ABCC4,EXO1,CDC25A*
	Tissue Development	5.41E-06	29	*DLGAP5,NME1,MKI67,AURKB,LMNB1,MAFG,STMN1,CDC25B,RAD51,HMOX1,EZH2,BRCA1,KIF2C,CDC25A,TACC3,PLK1,BUB1B,FOXM1,BIRC5,TPX2,CDK1,CCNB1,SKP2,CCNE1,E2F1,LAMA3,CD44,FEN1,CDK2*
	Connective Tissue Development and Function	1.78E-05	16	*PTTG1,DOT1L,RBL1,LMNB1,FANCD2,FASN,LIN9,E2F1,CENPA,BLM,BRCA1,RAD51AP1,CDK2,E2F2,EXO1,NUDT1*
	Lymphoid Tissue Structure and Development	2.70E-05	7	*CDC25B,FOXM1,AURKB,BIRC5,SKP2,CDC25A,CCNB1*

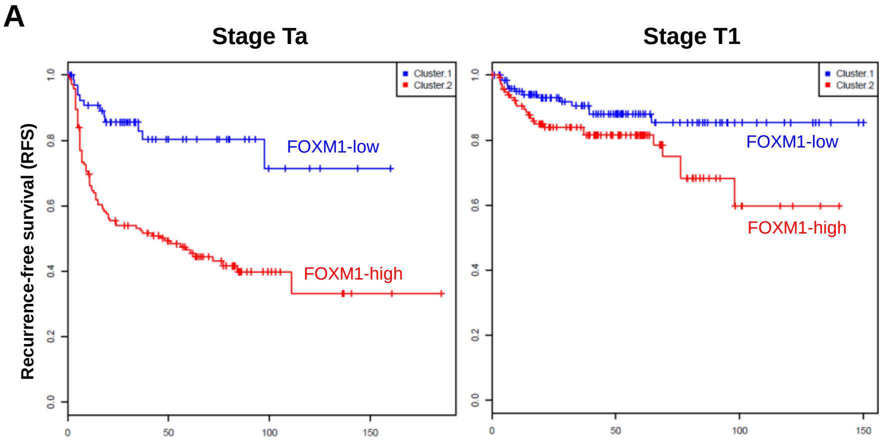

	# of Sample	Observed	Expected
**Cluster 1**	68	12	31.525
**Cluster 2**	143	80	60.475
**Result**	Chisq = 18.8832	df = 1	**p = 1.39e-05**

	# of Sample	Observed	Expected
**Cluster 1**	127	13	19.734
**Cluster 2**	120	25	18.266
**Result**	Chisq = 4.7998	df = 1	**p = 0.028**

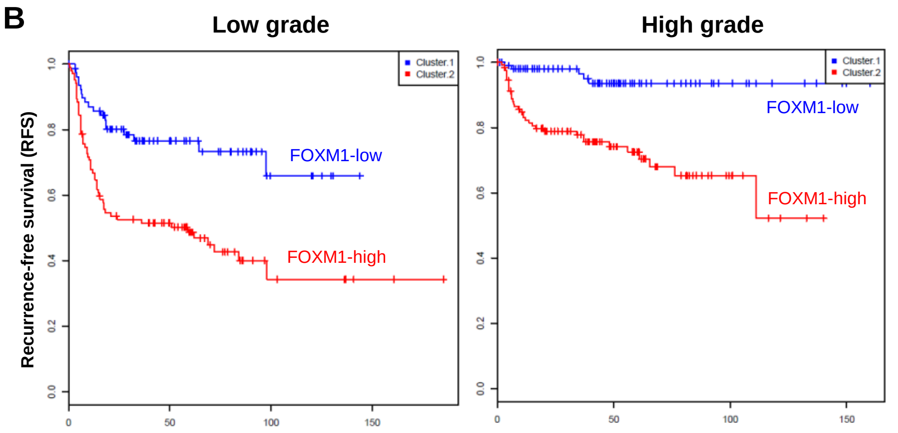

	# of Sample	Observed	Expected
**Cluster 1**	79	19	35.56
**Cluster 2**	103	56	39.44
**Result**	Chisq = 14.8516	df = 1	**p = 0.0001**

	# of Sample	Observed	Expected
**Cluster 1**	110	5	18.833
**Cluster 2**	130	35	21.167
**Result**	Chisq = 19.3608	df = 1	**p = 1.082e-05**

Figure S1. Prognosis according to tumor stage and grade of two subgroups divided by FOXM1 expression in NMIBC patient cohort. (**A**,**B**) The recurrence-free survival (RFS) curves comparing FOXM1-low and FOXM1-high expression groups for stratification of tumor stage as Ta, T1 and grade as Low, High.

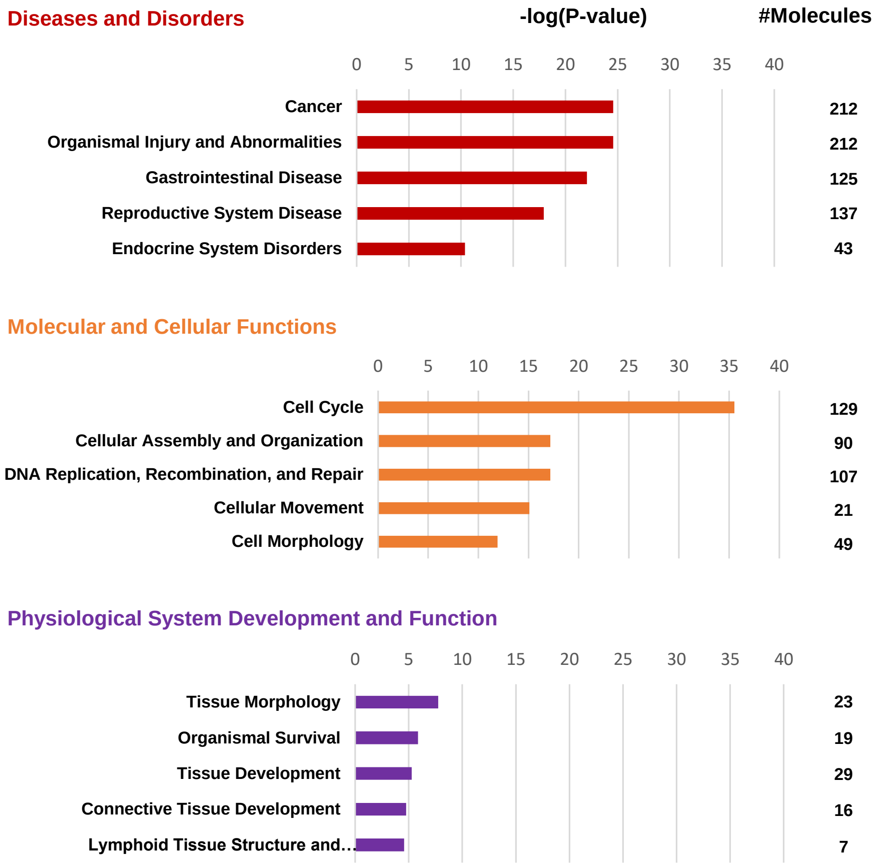

Figure S2. Function enrichment test and network analysis. In GSE13507 data, enrichment test and network analysis of 509 genes related to FOXM1 was confirmed by Ingenuity Pathway Analysis (IPA) analysis.

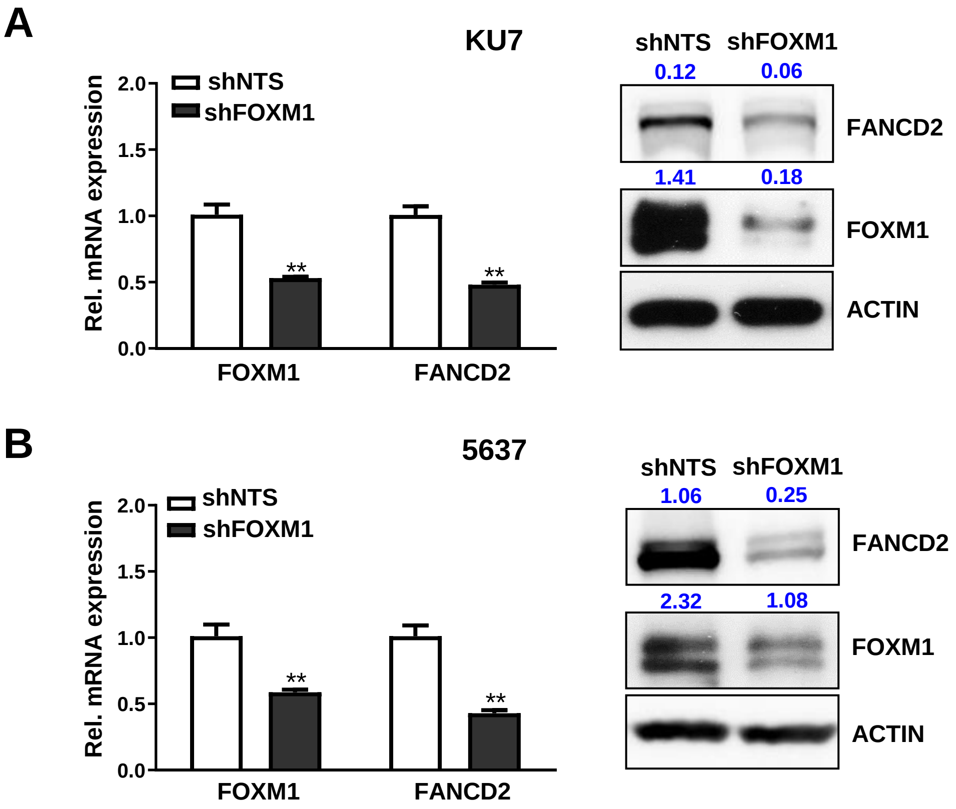

Figure S3. The expression of FOXM1 and FANCD2 on knockdown stable cell lines. (**A**,**B**) mRNA and protein levels of FOXM1, FANCD2 were measured by qRT-PCR and Western blotting in KU7 and 5637 cells stably expressing shNTS or shFOXM1. (**, $p < 0.01$;)

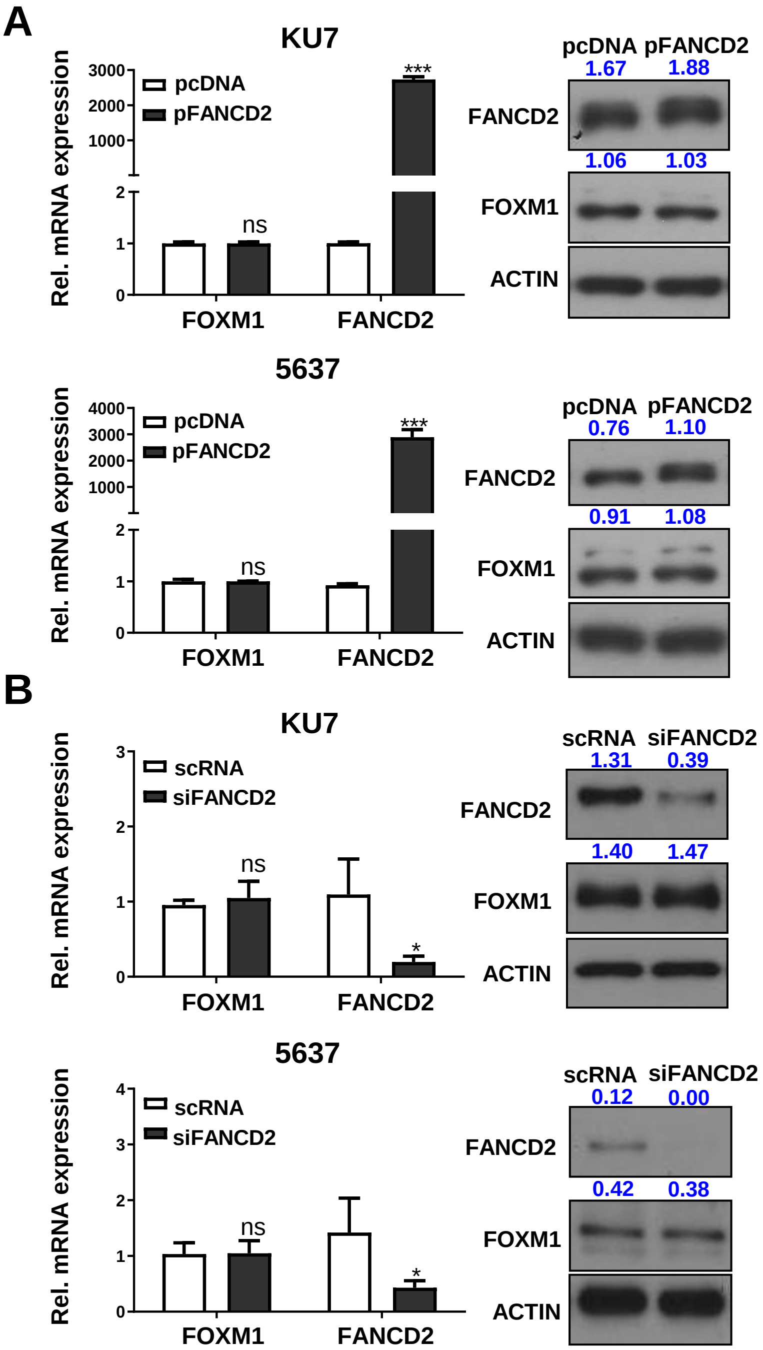

Figure S4. No significance of the expression of FOXM1 by overexpressed FANCD2. (**A**) pcDNA and pFANCD2 overexpression vector (1 μg/μL) transfected in KU7 and 5637 cells for 24 hours. (**B**) scRNA and siFANCD2 (100 nM) transfected in KU7 and 5637 cells for 24 hours. FOXM1 and FANCD2 mRNA and protein levels were determined by qRT-PCR and Western blotting. (*, $p < 0.05$; ***, $p < 0.001$;)

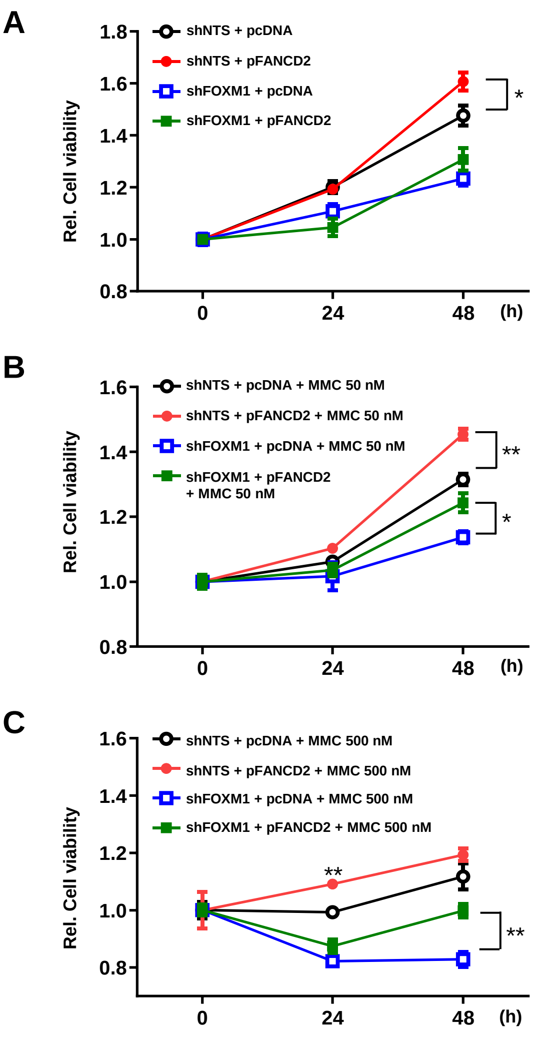

Figure S5. Increased cell viability due to overexpression of pFANCD2 in shFOXM1 cells. (**A**) shNTS and shFOXM1 cell lines were transfected with pFANCD2 and measured cell viability by MTT assay. (**B**,**C**) shNTS and shFOXM1 cells were transfected with pFANCD2 and measured survival rate of cells by MTT assay after exposure to the indicated 50 nM and 500 nM of MMC. (*, $p < 0.05$; **, $p < 0.01$)

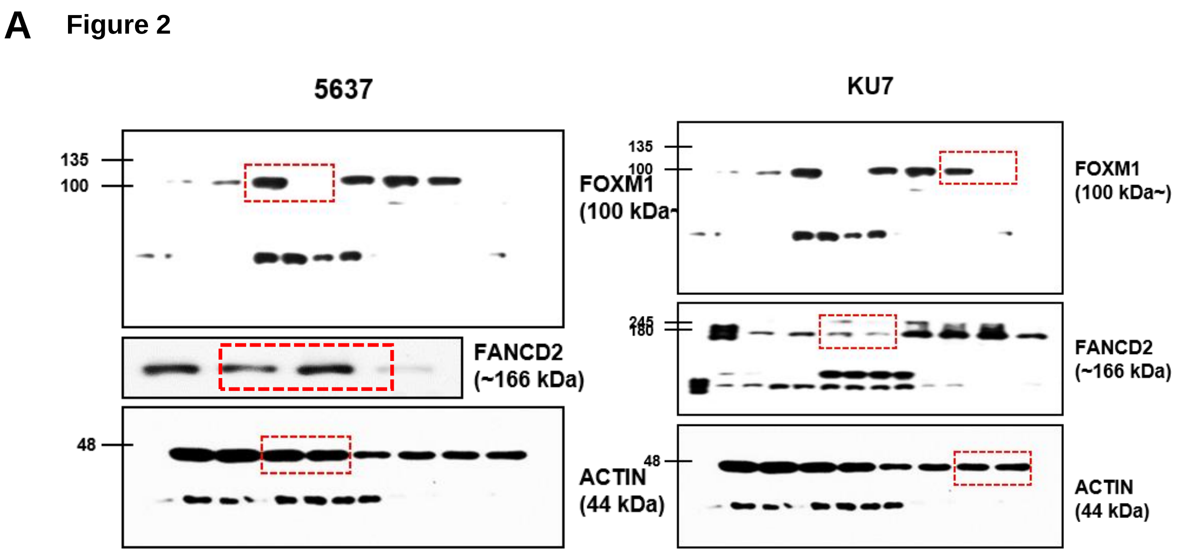

Figure S6. Uncropped blots showing all the western bands in 5637 and KU7 cell lines transfected with siFOXM1. For a clear comparison of FOXM1, FANCD2 expression following FOXM1 knockdown, crops were used in Figure 2.

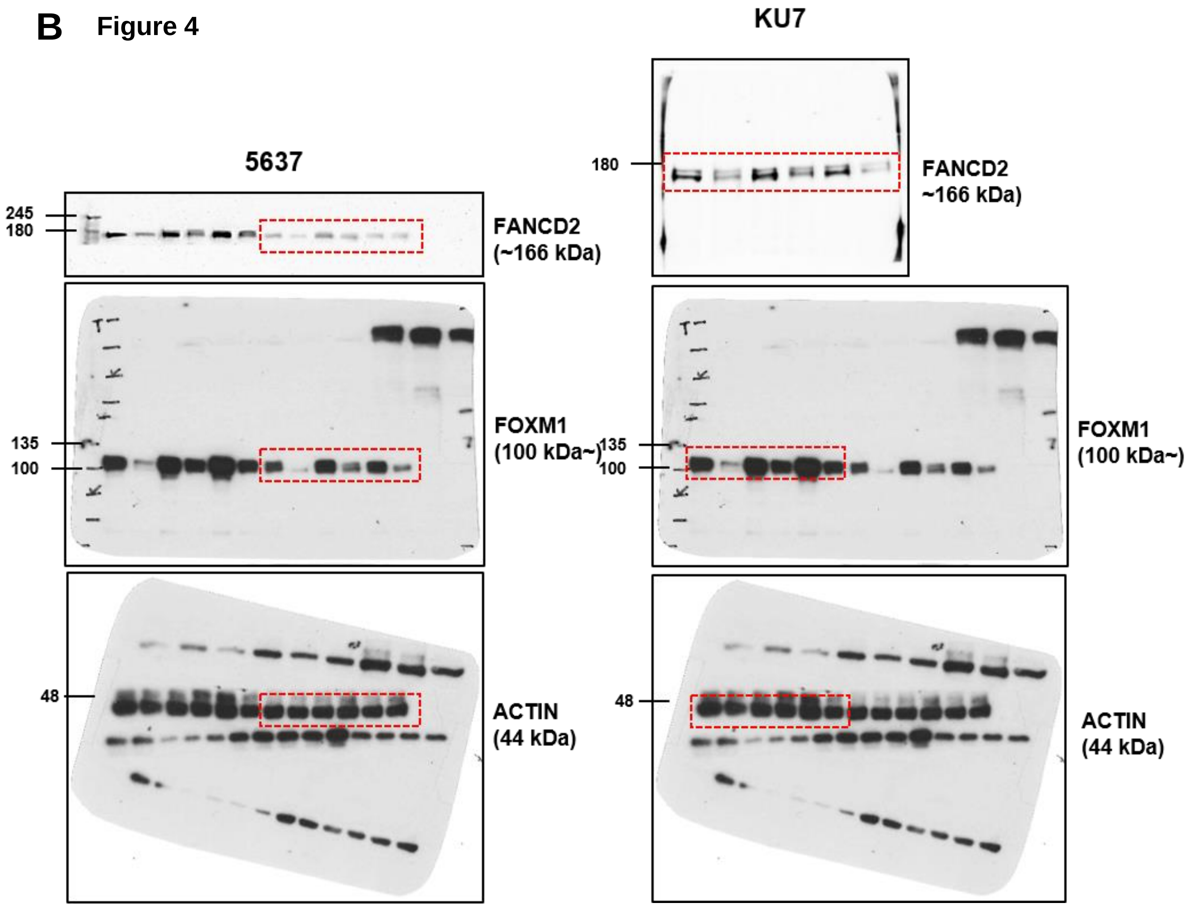

Figure S7. Uncropped blots showing all the western bands in 5637 and KU7 cell lines transfected with siFOXM1, treated to MMC. For a clear comparison of FOXM1, FANCD2 expression following FOXM1 knockdown, treated to MMC, crops were used in Figure 4.

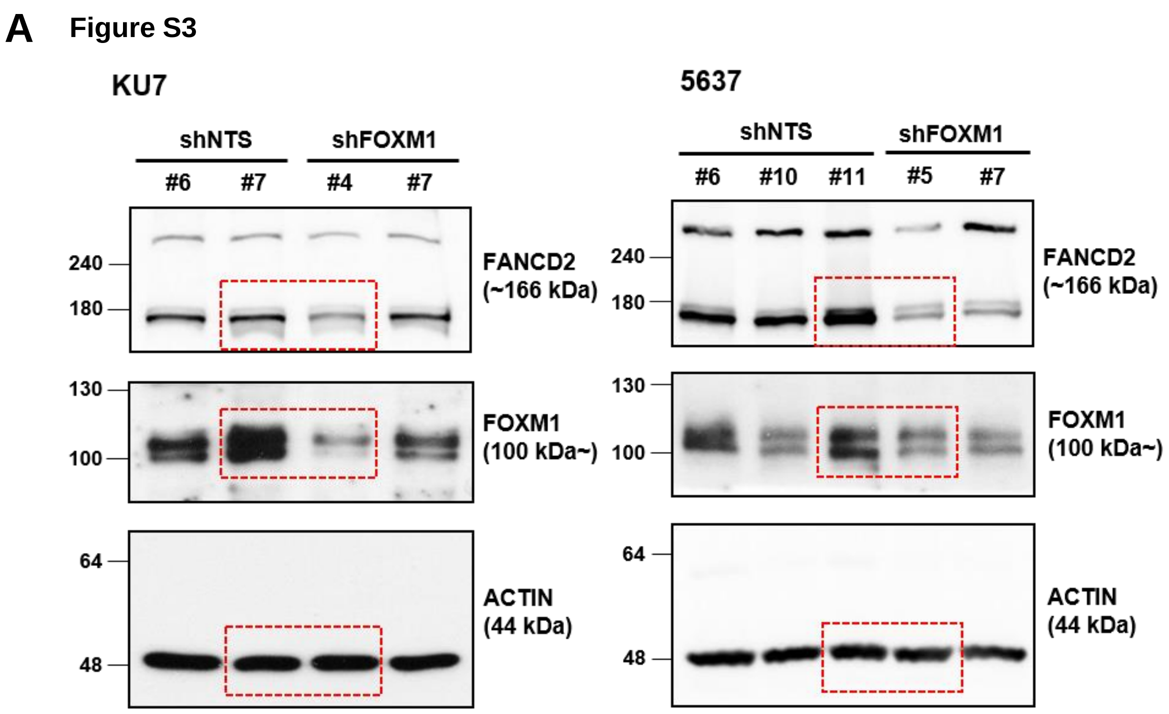

Figure S8. Uncropped blots showing all bands for stable cell line knocked down FOXM1 in KU7 and 5637. Crops were used in Figure S3 for a clear comparison of FOXM1, FANCD2 expression in the shFOXM1 cell line.

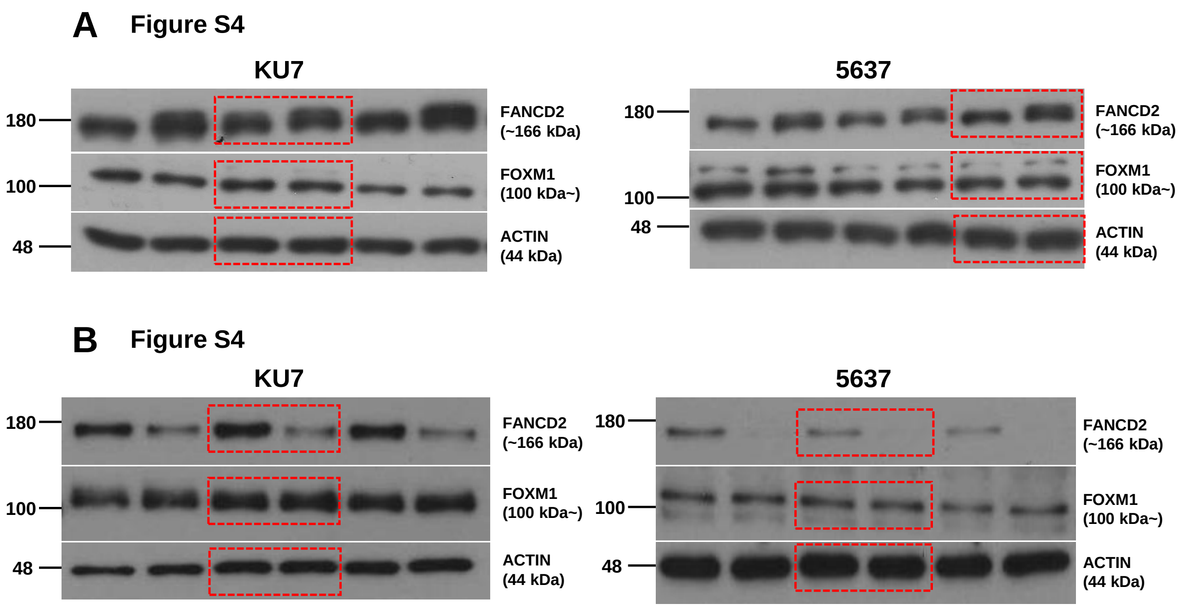

Figure S9. Uncropped blots showing all the western bands in 5637 and KU7 cell lines transfected with (**A**), pFANCD2 vector or (**B**), siFOXM1.
